# Health promoting Behaviors Among Adolescents: A Cross-sectional Study

**DOI:** 10.17795/nmsjournal14560

**Published:** 2014-04-17

**Authors:** Azra Sadat Musavian, Afsaneh Pasha, Seyyedeh-Marzeyeh Rahebi, Zahra Atrkar Roushan, Atefeh Ghanbari

**Affiliations:** 1 Department of Health (Community Health Nurse), Kashan University of Medical Sciences, Kashan, IR Iran; 2 Department of Nursing (Community Health Nurse), Guilan University of Medical Sciences, Rasht, IR Iran; 3 Department of Midwifery, Guilan University of Medical Sciences, Rasht, IR Iran; 4 Department of Biostatistics, Guilan University of Medical Sciences, Rasht, IR Iran

**Keywords:** Health Promotion, Lifestyle, Adolescent

## Abstract

**Background:**

Health maintenance and promotion are the fundamental prerequisites to community development. The best time for establishing healthy lifestyle habits is during adolescence.

**Objectives:**

Due to importance of health promotion behaviors in adolescents, this study was conducted to investigate health-promoting behaviors and its associated factors among high school students in Rasht, Iran.

**Materials and Methods:**

A cross-sectional descriptive study was conducted on 424 students during the first semester of the year 2012. We employed the multistage sampling design to recruit from private and public high schools in Rasht, Iran. The data collection instrument was a self-report questionnaire consisting of two parts. The first part of instrument was consisted of demographic questionnaire and the second part was adolescent health promotion scale (AHPS) questionnaire. AHPS questionnaire was consisted of six dimensions (nutrition, social support, health responsibility, life appreciation, physical activity, and stress management) to measure health promoting lifestyles. Statistical analysis was performed by SPSS 16 software employing ANOVA (analysis of variance) test, t-test, Mann-Whitney, and the Kruskal-Wallis.

**Results:**

The score of total Adolescent Health Promotion Scale were 3.58 ± 0.52 (possible range was 1-5). The highest score was in life appreciation dimension (3.99 ± 0.068) and the lowest score was in health responsibility dimension. Moreover, Significant associations were found between the adolescent health promotion Scale with age (P < 0.001), gender (P < 0.003), school grade (P < 0.011), father’s educational level (P < 0.045), mother’s educational level (P < 0.021), and mother’s occupation (P < 0.008).

**Conclusions:**

Female and older students are at higher risk of developing unhealthy lifestyle. Consequently, healthcare providers, health instructors, schoolteachers, and families must pay more attention to these students. Moreover, as most of lifelong healthy and unhealthy lifestyle habits are established during adolescence, developing effective health promotion and disease prevention strategies for adolescents seems crucial.

## 1. Background

Health maintenance and promotion are the fundamental prerequisites to the community development (1). Health promotion behaviors entail a positive approach to living and a means of increasing well-being and self-actualization (2). Health-promoting behaviors prevent diseases, decrease morbidities, improve the quality of life, and decrease healthcare costs (3). Accordingly, to determine individuals’ health status, such behaviors are usually examined (4). Currently, chronic non-communicable diseases are the first leading cause of disability and death worldwide (5). According to the estimations by world health organization, chronic non-communicable diseases will become the leading cause of 75% of all deaths in developing countries (6). These diseases are closely correlated with individuals’ lifestyle (7). A health-promoting lifestyle is a multi-dimensional pattern of self-initiated feelings and behaviors aiming at ensuring individual’s health, self-actualization, and self-accomplishment (8). Such lifestyle that includes eating a low-fat diet, regular physical activities, maintaining a healthy body weight, and avoiding smoking and stress, helps prevent many chronic diseases. Eating habit is the most important aspect of health promotion. Healthy diet plays an important role in the prevention of chronic diseases such as diabetes mellitus, hypertension, cardiovascular disease, and some types of cancers (9). Physical activity is also an important aspect of health promotion and a predictor of disability and death worldwide (10). The world health organization estimates that around two million deaths worldwide annually can be attributable to the lack of physical activity (11). Consequently, lifestyle modification is considered as a key disease prevention and health promotion strategy (12).

According to Wang et al. a good health-promoting behavior depends on the living habits adopted during early years of life (2). Maharaj also reported that half of the preventable premature deaths are associated with unhealthy habits developed in adolescence (13). Most of the healthy lifelong and unhealthy lifestyle habits are established during adolescence (1). Therefore health-promoting behavior among adolescents has become a research focus worldwide (2). Adolescence is the period of dynamic transition from childhood to adulthood and is associated with rapid changes in body, mind, and social relationships (2). There are an 1.2 billion adolescents, in other words, one in every five people, who live on the earth (14) Moreover, in countries like Iran, adolescents constitute a large part of the population in 2011; the Iranian 15- to 19-year-old population reached to 6607043 and in Guilan it has reached to 202829 (15). Therefore adolescents spend a great deal of their time at school (16), hence schools are appropriate places for health promotion educations (17).

Chen et al. (18) reported that to promote adolescents’ lifestyle, healthcare providers need to pay special attention to their eating habits, physical activity, social support, stress management skills, life appreciation, and health responsibility. Accordingly, they found that only 44.7% of American adolescents and 55.3% of Taiwanese adolescents had adopted a proper health promoting behaviors (18). Ortabag also reported that 44.3% and 56.5% of high school and guidance school students, respectively, practiced health-promoting behaviors (19). In Iran, Raiyat reported that junior high school students do health promotion behaviors occasionally (1). Health-related behaviors are influenced by many factors including social norms, culture, media, national health policies, advertisements, and environment (20). Although studies have shown adolescents health promotion behaviors are significantly related to male gender (1, 21, 22), there are also conflicting study results (12, 18, 19). Some studies indicated that younger adolescents more than older ones do health promotion behaviors (4, 22). However another studies results were sometimes contradictory (21). A few studies have shown that adolescents health promotion behaviors were significantly associated with the family monthly income (22). Recently few Iranian researchers have investigated the influences of some demographic characteristics on adolescent's health promotion behaviors (14). Nursing may play a key role in school health services. International directives highlight the important function of nurses in fulfillment of health promotion (19). Nurses and particularly community health nurses have key roles in education and spreading health promotion behaviors. Since health promotion viewpoints have recommended the population of educational centers, we conducted this study to investigate adolescents’ health promoting behaviors and its associated factors.

## 2. Objectives

Due to importance of health promotion behaviors in adolescents, this study was conducted to investigate health promoting behaviors and its associated factors among high school students in Rasht, Iran.

## 3. Patients and Methods

This was a cross-sectional descriptive study conducted in the first semester of the year 2012 in private and public high schools of Rasht, Iran. Sample size was estimated based on a previous study in which mean score health promotion behaviors was reported to be 2.50 (23). Then, it was estimated that 384 samples were needed based on the following parameters: α = 0.05 and sampling error of 1.9%; afterwards, 432 samples were selected for the study. However, 424 samples were entered in the final analysis because eight questionnaires were responded incompletely and were excluded. The inclusion criteria were studying at high schools located in urban districts of Rasht city, Iran, and having no diagnosed behavioral or psychological disorders according to their health records. We selected the students by using the multistage sampling design. At first, the city was divided into two areas, and the list of high schools in every area was determined. The schools were categorized based on the type of schools (boys or girls, private or public). Considering the type of schools, we randomly selected four schools from each area (eight schools totally). In every school, 18 students were selected randomly from every grade (the first through the third grades). Adolescent health promotion scale (AHPS) questionnaires were filled in the last 15 minutes of classes. The study instrument was a self-report questionnaire consisting of two parts. The first part of instrument was consisted of demographic questionnaire and the second part was adolescent health promotion Scale questionnaire. The demographic questionnaire included fifteen items about students’ personal information (age, gender, grade at school, birth rank, and history of taking medicine) and family background (father and mother’s educational and employment status, family average monthly income, family size and structure, type of residency, and source of acquiring health information). To examine the students’ health-promoting behaviors, we employed AHPS, which is based on the Pender's health promotion model and was developed by Chen for evaluating adolescent health promoting lifestyle (7, 18). The AHPS consists of a set of 40 items that assessed six dimensions of health-promoting behaviors including nutrition (six items), social support (seven items), health responsibility (eight items), life appreciation (eight items), physical activity (four items), and stress management (seven items). The AHPS obtains the frequency of reported behaviors by use of a five-point Likert scale: "never, rarely, sometimes, usually, and always" with a rating from on to five. To translate APHS from English to Farsi, we went through the translation-back-translation process. Accordingly, we invited 16 nursing faculties to assess the content validity index. We revised the Farsi version of AHPS according to their comments. Then, we administered the scale to 20 students and used their data to assess the reliability of the scale by checking its internal consistency. The reliability coefficient for the total scale and each dimension of nutrition, social support, health responsibility, life appreciation, physical activity, and stress management were calculated using Cronbach’s alpha coefficients as 0.90, 0.78, 0.64, 0.81, 0.78, 0.75 and 0.74, respectively. One of the items in social support, which had greatly decreased the Cronabch’s alpha, was deleted from the scale. Accordingly, the final version of AHPS included 39 items. Consequently, the total score of the scale ranged from 39 to 195. However, to maintain the 1-5 metric of item scores and to be able to compare the dimensions with each other, we calculated the mean, instead of sums, for the total score of AHPS and its dimensions. Accordingly, the possible range of the scores of AHPS and its dimensions were 1-5.

### 3.2. Data Analysis

We employed the statistical package for social sciences (SPSS v. 16.0; SPSS Inc. Chicago, USA) for data analysis. We analyzed the distribution of the study variables using the kolmogrov-smirnov test. Accordingly, we analyzed overall health promotion behaviors scale of the normally distributed variables using the one-way analysis of variance (one-way ANOVA) and the independent samples t-tests. In addition, six dimensions of health promotion behaviors variables having non-normal distribution were analyzed using the Mann-Whitney and the Kruskal-Wallis tests. A P-value lower than 0.05 was considered as statistically significant level in all the tests.

### 3.3. Ethical Considerations

The study was approved by the Ethics Committee of Research Deputy of Guilan University of Medical Sciences. Aims of the study were explained to the participants and they were assured of the confidentiality of personal information before starting the study and all signed a written informed consent. The researchers conformed to all the ethical issues in accordance with the Helsinki declaration.

## 4. Results

A total of 424 students participated in the study of which 216 (50.9%) were female and 208 (49.1%) were male. The mean age of high school students was 15.97 ± 0.97, which ranged from 14 to 18 years of age. About 64.1% of the students’ fathers were self-employed and about 80.6% of their mothers were housewives. In addition, the majority of the students reported that they had no health instructor (64.7%) (Table 1). 

**Table 1.** The Distribution of Some of Socio-Demographic Variables

Demographic Variables	No. (%)
**Gender**	
Male	208 (49.1)
Female	216 (50.9)
**Birth rank**	
1	210 (49.8)
2	130 (30.8)
3	46 (10.9)
≥ 4	36 (8.5)
**Medication**	
Yes	383 (91)
No	38 (0.9)
**Family monthly income, $**	
< 200	115 (27.1)
200-400	175 (41.3)
> 400	125 (29.5)
**Family size**	
3–4	306 (72.2)
5-6	100 (23.6)
≥ 7	17 (4)
**House type**	
Rented	70 (16.6)
Private	335 (79.6)
Other	16 (3.8)
**Family structure**	
Father or mother or none	21 (5)
Both	402 (95)
**Source of health information**	
TV or radio	241 (56.8)
Internet	146 (34.4)
Newspaper or book	34 (8)

Study findings revealed that the mean and the standard deviation of total score of AHPS were 3.58 ± 0.52 (possible range was 1-5). The highest through lowest mean scores were seen consecutively in life appreciation (3.99 ± 0.67), nutrition (3.71 ± 0.64), stress management (3. 54 ± 0.76), social support (3.50 ± 0.75), physical activity (3.39 ± 1.10), and health responsibility dimensions (3.26 ± 0.75). The results of the independent-samples t-test showed that the health promotion mean score of male students was significantly higher than the mean score of female students (P = 0.003; Table 2). Moreover, the results of this test revealed that regarding health promoting behaviors, students who had a history of medication therapy did not differ significantly from those who had not such a history (P = 0.625). Similarly, we found no statistically significant difference between the mean score of students with and without health instructor (P = 0.845). Study findings revealed that the mean score of students’ health promotion living with their both parents was higher than the mean score of students living with either one or none of their parents; however, the results of the independent-samples t-test indicated that this difference was not statistically significant (P = 0.066). On the other hand, the results of the one-way ANOVA test showed that students in different school grades differed significantly in terms of health promotion behaviors (P = 0.011; Table 2). The results of the Tukey’s post-hoc test revealed that this difference was between first and third grade students. Moreover, the results of the one-way ANOVA test indicated that students in different age groups differed significantly in terms of health promotion behaviors (P = 0.001) (Table 2). In other words, older students had lower health promotion score. This finding implies that students’ health promotion score has a significant inverse association with their age.

**Table 2.** Distribution of Variables Associated With Total Health Promotion Behaviors Among Adolescents ^a^

Variable	No. (%)	Mean ± SD	Test	P Value
**Age**				0.001
14	25 (5.9)	3.72 ± 0.47	ANOVA	
15	177 (27.6)	3.71 ± 0.56		
16	114 (34)	3.58 ± 0.51		
17	121 (28.5)	3.48 ± 0.46		
18	17 (4)	3.25 ± 0.72		
**Gender**				0.003
Male	208 (49.1)	3.67 ± 0.50	t-test	
Female	216 (50.9)	3.51 ± 0.54		
**School grade**				0.011
1	142 (33.5)	3.67 ± 0.56	ANOVA	
2	140 (33)	3.61 ± 0.48		
3	142 (33.5)	3.48 ± 0.52		
**Father’s education level**				0.045
Illiterate and Diploma >	120 (28.6)	3.55 ± 0.43	ANOVA	
Diploma	175 (41.8)	3.60 ± 0.54		
University	124 (29.6)	3.66 ± 0.56		
**Mother’s education level**				0.021
Illiterate and Diploma >	131 (31)	3.59 ± 0.38	ANOVA	
Diploma	203 (48.1)	3.55 ± 0.57		
University	88 (20.9)	3.73 ± 0.47		
**Mother’s job**				0.08
Care provider	10 (2.4)	3.93 ± 0.54	ANOVA	
Employee	44 (10.4)	3.78 ± 0.43		
Homemaker	340 (80.6)	3.56 ± 0.51		
Self-employed and other	28 (6.6)	3.43 ± 0.71		

^a^ Abbreviation: ANOVA, analysis of variance.

We found that the students’ health promotion score did not significantly associate with their fathers’ job (P = 0.648). However, according to the results of the one-way ANOVA test, the students’ health promotion score was significantly associated with their mother’s job (P = 0.008) (Table 2). As the Tukey’s post-hoc test did not detect this difference, we used the least significant difference (LSD) post-hoc test in this case. The results of this test revealed that the mean health promotion score of students whose mothers were healthcare providers or employee was significantly higher than the mean score of students whose mothers were housewives or self-employed. The results of the one-way ANOVA test also revealed that the students’ mean health promotion score was significantly associated with their fathers’ and mothers’ educational status (P = 0.045 and 0.021, respectively) (Table 2). The results of the LSD post-hoc test revealed that the mean health promotion score of students whose parents had university education was higher than the mean score of other students. On the other hand, the results of the one-way ANOVA test indicated that students’ health promotion mean score did not significantly associate with family size (P = 0.055), house type (P = 0.055), family monthly income (P = 0.140), birth rank (P = 0.235), and source of acquired health information (P = 0.359). In terms of the AHPS dimensions, the results of the Mann-Whitney test revealed that male students’ mean scores of the nutrition, life appreciation, and physical activity dimensions were higher than the female students’ mean scores (P < 0.05; Table 3). Moreover, the results of this test showed that female students’ mean score of health responsibility dimension was significantly higher than male students’ scores (P = 0.042). On the other hand, according to the results of the Kruskal-Wallis test, the mean score of students in different school grades also has significant difference in nutrition, health responsibility, life appreciation, and physical activity dimensions (P < 0.05; Table 3). Social support and stress management have no significant difference with the school grade (Table 3). 

**Table 3.** Dimensions Score of Adolescent Health Promotion Scale With Gender and School Grade ^a^, ^b^

Variables	Gender			School Grade			
	Male	Female	P Value	1st	2nd	3rd	P Value
**Nutrition**	3.90 (0.52)	3.54 (0.69)	< 0.001	3.67 (0.63)	3.83 (0.58)	3.64 (0.68)	0.042
**Social support**	3.45 (0.78)	3.54 (0.72)	0.185	3.49 (0.71)	3.52 (0.81)	3.47 (0.74)	0.837
**Health responsibility**	3.18 (0.76)	3.33 (0.73)	0.042	3.37 (0.79)	3.32 (0.70)	3.09 (0.73)	0.009
**Life appreciation**	4.08 (0.60)	3.90 (0.73)	0.022	4.09 (0.70)	4.01 (0.65)	3.87 (0.66)	0.009
**Physical activity**	3.66 (1.06)	3.13 (1.08)	< 0.001	3.65 (1.05)	3.41 (1.08)	3.10 (1.11)	< 0.001
**Stress management**	3.57 (0.71)	3.51 (0.81)	0.605	3.60 (0.89)	3.53 (0.72)	3.47 (0.66)	0.121

^a^ P value is the results of the Mann-Whitney test.

^b^ All data are presented in Mean±SD.

## 5. Discussion

A few studies concerning health promotion behaviors by AHPS questionnaire in Iranian adolescents have been performed; therefore aim of this study was to investigate adolescents’ health-promoting behaviors by AHPS questionnaire and its associated factors. Study findings revealed that the students’ mean of AHPS total score was 3.58 ± 0.52. This finding implies that students usually tend to display health-promoting behaviors. Ortabag et al. also reported the similar findings (19). However, Raiyat by using HPLPII instrument found that junior high school students exhibited health promoting behaviors occasionally (1). These slight differences might be attributed to the sample size and instrument. We found that high school students did poorer at the health responsibility dimension of AHPS. This finding is in line with the findings of previous studies (1, 4). Raiyat noted that adolescents are too young to understand their role in promoting their health and quality of life. They also reported that very few students know how to perform and engage in self-care activities. However, Ortabag reported that students did poorer at the physical activity dimension (19). Furthermore, another study in Iran indicated that Iranian’s adolescents have a sedentary lifestyle that might be due to spending too much time watching television and playing computer games, as well as due to decreased opportunities for exercise in schools and communities (14, 23).

The highest scores belonged to the life appreciation dimension. This is in line with the findings of other studies (2, 14). Probably, satisfaction of their life contributed to this finding in adolescents (14). The study findings revealed that the mean of male students’ health promotion score was significantly higher than the mean of female students’ score. However, Ortabag and Chen found that female students mean of health promotion score was higher than male students’ score (18, 19).

It might be attributed to the difference in sample size. We also found that male student’ mean scores of the nutrition, life appreciation, and physical activity dimensions were significantly higher than the female students’ mean scores. However, previous studies reported conflicting results. For example, Raiyat, Wang, Aghamolaei, and Ortabag reported that male students’ performance on the physical activity dimension was better than the female students’ performance (1, 2, 14, 19). On the other hand, Aghamolaei and Chen et al. found that female students’ mean score of life appreciation dimension was higher than the male students’ score (14, 18). Such controversies are probably due to the effects of contextual factors on the association of health promoting behaviors and students’ gender. We also found that the female students’ mean score of the health responsibility dimension was greater than the male students’ score. Other studies reported the same finding (4, 12, 14). We believe that comparing to the male students, female students are more attentive to health promoting behaviors. However, the present study revealed no difference between the genders with regard to social support and stress management whereas a previous study showed that females were more confident with regards to the social support dimension (2). Our study also showed that scores of nutrition, health responsibility, life appreciation, and physical activity dimensions were significantly with grade. Chen et al. also acquired the similar findings regarding life appreciation (18). The findings of the study revealed that health promotion was inversely associated with students’ age and school grade. Chen et al., Wang et al. and Ortabag et al. also reported the same findings (2, 18, 19). In Iran, older students’ are intensely involved in the training courses of university entrance exam and hence, they are too busy to pay attention to health promoting behaviors. We also found that students’ mean score of health promotion was positively associated with their mothers’ educational status. Ay et al. and Chen et al. also reported that students’ mean score of health promotion has a positive association with parents’ educational status (18, 21). This finding is probably because educated parents have more health information and hence, pay more attention to their children’s health promoting behaviors. Another finding of the study was that students’ health promotion was significantly associated with their mothers’ job. Accordingly, the AHPS total score of students who mothers were either healthcare providers or employee were higher than the score of other students. Can also reported the same finding (24). It seems that working mothers probably have more health information and help their children adopt a healthy lifestyle. 

In conclusion, Students affiliated to the high schools located in Rasht, Iran, usually engaged in health promoting behaviors. However, further intervention is needed to improve their health responsibility. Moreover, as female students’ AHPS mean score was significantly lower than the male students’ score, healthcare providers, health instructors, schoolteachers, and families need to pay more attention to female students’ health promoting behaviors. On the other hand, older students, who are intensely involved in preparatory courses for gaining admission into the highly competitive Iranian university entrance exam and hence, are too busy to engage in health promoting behaviors, need careful attention. Generally, as most of the lifelong healthy and unhealthy lifestyle habits are established during adolescence, developing effective health promotion and disease prevention strategies for adolescents seems crucial. In Addition, further studies with AHPS instruments are recommended to assess these behaviors and its associated factors in adolescents. Moreover, the main limitations of this study were Students’ health status and psychological state at the time of study that might had affected their responses to the study questionnaire.
